# Dietary Soybean Oligosaccharides Addition Increases Growth Performance and Reduces Lipid Deposition by Altering Fecal Short-Chain Fatty Acids Composition in Growing Pigs

**DOI:** 10.3390/ani13233648

**Published:** 2023-11-25

**Authors:** Shanchuan Cao, Juan Wang, Jianfei Zhao, Shuwei Li, Wenjie Tang, Hui Diao, Jingbo Liu

**Affiliations:** 1 School of Life Science and Engineering, Southwest University of Science and Technology, Mianyang 621010, China; 2 Department of Animal Resource and Science, Dankook University, Cheonan 31116, Republic of Korea; 3 Animal Breeding and Genetics Key Laboratory of Sichuan Province, Sichuan Animal Science Academy, Chengdu 610066, China; 4 Livestock and Poultry Biological Products Key Laboratory of Sichuan Province, Sichuan Animtech Group Co., Ltd., Chengdu 610066, China

**Keywords:** growth performance, meat quality, carcass traits, lipid metabolism, growing–finishing pig

## Abstract

**Simple Summary:**

In this study, we investigated the effects of adding soybean oligosaccharides (SBOS) to growing–finishing pig diets on growth performance, carcass traits, meat quality, and fat deposition. SBOS are considered to be one of the causes of diarrhea in piglets. However, their impact on growing and fattening pigs has not been reported. The results of this study showed that adding 0.8% SBOS to the diet of growing–finishing pigs can increase the average daily weight gain. Hindgut fermentation of SBOS, which alters the composition of short-chain fatty acids, was the mechanism behind the reduction in fat deposition in growing–finishing pigs.

**Abstract:**

One hundred and twenty-eight boars and gilts of the Duroc × Landrace × Yorkshire variety with an initial body weight (BW) of 52.49 ± 0.48 kg were used in a randomized complete block design for a 63-day experiment. The four treatment groups were: control diet (CON), CON + 0.2% soybean oligosaccharides (SBOS), CON + 0.4% SBOS, and CON + 0.8% SBOS. The results showed that the average daily weight gain (ADG) was significantly higher in the 0.8% SBOS group than in the CON group on days 0–63 (*p* < 0.05). Compared with the CON group, adding 0.8% SBOS to the diet significantly increased the carcass weight, dressing percentage, and carcass lean percentage, but decreased the average backfat depth of growing–finishing pigs (*p* < 0.05). Adding different concentrations (0.2%, 0.4%, and 0.8%) of SBOS to the diet can significantly increase the concentrations of acetate, propionate, and butyrate in feces (*p* < 0.05). The activities of malic enzyme and fatty acid synthase in the 0.8% group were significantly lower than those in the 0.2% and CON groups (*p* < 0.05). In summary, 0.8% SBOS supplementation to growing–finishing pigs’ diets can reduce lipid deposition and increase ADG.

## 1. Introduction

Soybean oligosaccharides (SBOS) are the major carbohydrates contained in soybean meal. A previous study has shown that SBOS had prebiotic properties due to their ability to regulate intestinal microbial structure and metabolism and were considered safe and reliable materials [1]. However, monogastric animals lack endogenous enzymes to digest SBOS, so SBOS are difficult to utilize for monogastric animals. Research on SBOS in pigs is very limited. Previous studies on piglets showed that a high concentration of SBOS (1%) could cause intestinal disorders and diarrhea in piglets [2]. The digestibility of organic matter and crude protein (CP) decreased by 25% in piglets fed high concentrations of SBOS soybean hulls [3]. The addition of SBOS to the diet of growing pigs reduced the digestibility of nitrogen and amino acids [4]. The above studies have shown that SBOS play the role of anti-nutritional factors in the diet of pigs. An in vitro fermentation test using Huanjiang mini-pigs’ colon digesta showed that SBOS can improve the balance and metabolism of colonic flora [5]. A study conducted on mice demonstrated that the intake of soybean oligosaccharides led to an increase in the count of advantageous gut microbes, which consequently enhanced the immune system of the mice [6]. In addition, studies have shown that short-chain oligosaccharides that cannot be digested by upper gastrointestinal digestive enzymes can be selectively fermented by certain bacteria in the large intestine [7,8]. We speculate that pigs can utilize SBOS through hindgut microbial fermentation. Diarrhea caused by SBOS in piglets may be due to the limited fermentation capacity of the hindgut. However, the effect of SBOS on growth performance and other indicators of finishing pigs has not yet been reported. We hypothesized that growing–finishing pigs can efficiently utilize SBOS. Therefore, the aim of this study was to investigate the effects of dietary SBOS supplementation at different concentrations on growth performance, carcass characteristics, meat quality, and fat deposition in growing–finishing pigs.

## 2. Materials and Methods

### 2.1. Experiment Design, Animals, and Environment

One hundred and twenty-eight boars and gilts of the Duroc × Landrace × Yorkshire variety with an initial body weight (BW) of 52.49 ± 0.48 kg were employed in a randomized complete block design for a 63-day experiment. Pigs were randomly divided into four treatment groups based on BW and sex before the experiment. Each dietary treatment had eight replicates with four pigs per replicate (two boars and two gilts). The four treatment groups were: control diet (CON), CON + 0.2% SBOS, CON + 0.4% SBOS, and CON + 0.8% SBOS. All of the pigs utilized in the experiment originated from New Hope Beichuan New Changle Agricultural Husbandry Co. SBOS were purchased from Mianyang Heben Bioengineering Co., Ltd. (Mianyang, China). The basic diet was divided into two phases according to National Research Council (2012) recommendations [9]. The basal diets for the two different phases are shown in Table 1. The form of feed was powder. On day 21, the pigs were moved from the growing house to the finishing house, and the diet was changed. For the duration of the experiment, all the pigs were housed in plastic floor pens and had free access to food and water. Each pen in the growing and finishing pig houses was equipped with a semi-automatic feed trough and a nipple drinker. The size of the growing pig pen was 1.1 m × 2.1 m. The size of the finishing pig pen was 1.5 m × 3.1 m. The temperature and humidity of the growing house throughout the experiment were 20–22 °C and 60%, respectively. The temperature and humidity of the finishing house throughout the experiment were 19–21 °C and 60%, respectively.

### 2.2. Growth Performance and Sample Collection

The BW for each pig was measured to determine average daily gain (ADG) at 8 a.m. on days 0, 21, 42, and 63. Daily amounts of feed and residual feed in each pen were recorded to determine the average daily feed intake (ADFI) and feed-to-gain ratio. Chrome trioxide (0.5%) was added as an endogenous indicator during the last week (days 57 to 63) of the experiment. Fecal samples were obtained via rectal massage of pigs in each pen from 6 a.m. on day 62 to 6 a.m. on day 63 to determine digestibility values of dry matter (DM), CP, ether extract (EE), and gross energy (GE). At 7 a.m. on day 63, jugular venous blood was collected from one pig per pen to assess blood components, with a male-to-female ratio of 1:1 in each treatment group. After weighing, the blood-collected pigs were slaughtered via jugular exsanguination after electronarcosis. After slaughter, the carcass weight and length were measured. From the carcass weight and the slaughter weight, the dressing percentage was calculated. The average backfat thickness of all pigs was calculated by measuring at three different sites (shoulder, mid-back, and loin) 5 cm to the right of the midline, just above the point of the elbow, last rib, and last lumbar vertebra. A small amount of backfat sample was collected to measure fat cell diameter and lipid metabolism-related enzyme activity. The lean meat percentage was calculated by dividing the left carcass after slaughtering into lean meat, fat, skin, and bones. A portion of the longissimus dorsi muscle (LDM) was harvested to determine meat quality. Colonic contents were collected to determine short-chain fatty acid (SCFA) concentration.

### 2.3. Nutrient Digestibility and Blood Profile Analysis

Collected feces and feed samples were dried in a ventilated oven at 65 °C for 2 days. The dried samples were crushed and passed through a 1 mm sieve. The method used herein was adapted from Liu et al. to determine the concentration of calcium and phosphorus in the diet [10]. The content of DM was determined by drying the sample at 105 °C for 2 h. CP concentration was determined using the Leco CHNS-932 analyzer combustion method (Leco Corp., St. Joseph, MI, USA). EE was determined using the Soxhlet extraction method. GE in dietary and fecal samples was analyzed using a fully automated calorimeter (BYLRY-3000W, Beijing Grand Boyu Technology. Co., Ltd., Beijing, China). Fecal and feed samples were treated with concentrated nitric acid and perchloric acid, and the absorbance of the digestion solution at 450 nm was measured to determine the concentration of Cr_2_O_3_ [11]. The components in the blood were measured using a hematology analyzer (BK-600, biobased, Jinan, China).

### 2.4. Meat Quality Analysis

The LDM muscle pH at 45 min and 24 h was determined using a pH meter (pH-STAR; SFK-Technology, Herlev, Denmark). Determination of L*, a*, and b* values of LDM was accomplished using a colorimeter CR-300 (Minolta, Osaka, Japan). Dripping loss was assessed after the suspension of LDM at 4 °C for 24 h. The LDM was vacuum-packed and then placed in a 70 °C water bath to measure the cooking loss. The shear force of LDM was measured using a texture analyzer (TA.XT. plus; Stable Microsystems, Surrey, UK) [12].

### 2.5. SCFAs Analysis

Initially, 15 g of colon contents was weighed; they were then mixed with 15 mL of distilled water and centrifuged at 4000× *g* and 18 °C for 5 min. From the supernatant, 5 mL was extracted. Then, an equal proportion of HCl was added and the solution was mixed. Then, the solution was submitted to centrifugation at 14,000× *g* and 17 °C for 10 min. Afterward, the supernatant was extracted and injected into a gas chromatograph coupled to a flame ionization detector. The column contained 10% SP 1200, 1% H3PO4 and acid-washed 80/100 Chromosorb W (length 1.8 m) with stationary phase SP 1200 (Supelco, PA, USA). Nitrogen was used as the carrier gas.

### 2.6. Lipid Metabolism Analysis

Backfat was extracted using methanol–chloroform to determine lipid content. Back fat was stained with hematoxylin and eosin to measure the diameter of adipocytes. The adipose tissue was embedded in paraffin and sectioned. After dehydration to remove paraffin and water, the tissue was stained with hematoxylin and eosin. Subsequently, the tissue samples were microscopically photographed and the diameter of adipose cells was measured [13]

Lipogenic enzyme activity method: first, a mixed solution of 0.25 mol/L frozen sucrose, 1 mmol/L EDTA, and 1 mmol/L dithiothreitol was prepared. Then, 500 mg of backfat was placed into the mixed solution and homogenized. Then, the mixture was centrifuged at 10,000× *g* and 4 °C for 1 h. The supernatant was taken to measure the activities of malic enzyme (ME) and glucose-6-phosphate dehydrogenase (G-6-PDH) at 340 nm absorbance at 25 °C, and the activity of fatty acid synthase (FAS) at 340 nm absorbance at 28 °C [14,15].

### 2.7. Statistical Analysis

The soy oligosaccharide level added to the diet was evaluated through ANOVA analysis using SAS’s GLM procedure (SAS Inst. Inc., Cary, NC, USA) with a randomized complete block design. Growth performance and nutrient digestibility were analyzed using the pen as the experimental unit. Blood profile, carcass quality, meat quality, SCFA, and lipid metabolism were analyzed using the individual pig as the experimental unit. Results with *p* < 0.05 were considered statistically significant.

## 3. Results

### 3.1. Growth Performance, Nutrient Digestibility, and Blood Profile

The average BW on day 63 and the ADG from days 0–63 of the group receiving 0.8% SBOS were significantly greater than those of the CON group (Table 2, *p* < 0.05). However, we did not observe significant differences in ADFI, feed-to-gain ratio (Table 2, *p* > 0.05), and nutrient digestibility (Table 3, *p* > 0.05) among treatments. Compared with the CON group, the concentration of HDL-C in the blood in the 0.8% SBOS group was significantly increased (Table 4, *p* < 0.05). There were no significant changes in blood LDL-C, GLU, T-CHO, and TG concentrations among treatment groups (Table 4, *p* > 0.05).

### 3.2. Carcass Traits and Meat Quality

Compared with the CON group, adding 0.8% SBOS to the diet significantly increased the carcass weight, dressing percentage, and carcass lean percentage, but decreased the average backfat depth of growing–finishing pigs (Table 5, *p* < 0.05). Feeding 0.4% SBOS increased the carcass weight of growing–finishing pigs compared with the CON group (Table 5, *p* < 0.05). In addition, there was no significant effect on the meat quality of growing–finishing pigs when feeding them different concentrations of SBOS (Table 6, *p* > 0.05).

### 3.3. SCFAs and Fat Deposits

Adding different concentrations (0.2%, 0.4%, and 0.8%) of SBOS to the diet can significantly increase the concentrations of acetate, propionate, and butyrate in feces compared with the CON group (Table 7, *p* < 0.05). The concentrations of acetate, propionate, and butyrate in the feces were higher in the 0.8% SBOS group than in the 0.2% SBOS group and the 0.4% SBOS group (Table 7, *p* < 0.05). The concentrations of SBOS had no effects on adipose tissue lipid content, fat cell diameter, and G-6-PDH activity (Table 8, *p* > 0.05). The activities of ME and FAS in the 0.8% group were significantly reduced compared with the 0.2% SBOS and CON groups (Table 8, *p* < 0.05).

## 4. Discussion

### 4.1. Growth Performance, and Nutrient Digestibility

More economical growth performance is the first goal pursued by agricultural and animal husbandry farmers. Growth performance denotes comprehensive performance under the combined effect of multiple factors, such as variety, feed nutritional levels, minerals such as calcium and phosphorus, functional additives, and feeding and management levels [16,17,18,19,20,21,22,23]. Previous studies in piglets have shown that dietary supplementation with soy oligosaccharides reduced growth performance and induced diarrhea [2,24]. Zhang et al. found that adding 1% (weight gain 0.25 vs. 0.21) and 2% (weight gain 0.25 vs. 0.17) stachyose to piglet diets slowed the growth rate [2]. Another study showed that reducing the addition of SBOS in piglet diets reduced diarrhea (the duration of diarrhea was 6 and 2.4 days for soybean meal and soy protein concentrate based diets, respectively) and improved growth performance (the growth rate for a diet based on soy protein concentrate and soybean meal was 244 and 224 g/day, respectively) [24]. Adding soybean extract containing stachyose and raffinose to piglet diets reduced the digestibility of organic matter, nitrogen free extract, and CP by 20% [3]. In addition, supplementation of SBOS such as stachyose and raffinose was shown to reduce nitrogen (81.4 vs. 75.9) and amino acid (83.8 vs. 79.0) digestibility in studies related to growing pigs [4]. The above results were due to the digestion of SBOS changing the penetration difference between the mucosa and plasma, causing digestive tract disorders and increasing the risk of diarrhea in weaned piglets. The study results demonstrated that the inclusion of SBOS in feed had no impact on the digestibility of nutrients (DM, CP, EE, and GE). The difference between this and previous studies may be because the fermentation capacity of intestinal microorganisms increased after growing and fattening pigs, and the intestinal microorganisms fermented SBOS and then changed their anti-nutritional properties, which did not affect intestinal function and led to a decrease in nutrient digestibility. Adding 0.8% SBOS could increase the average BW on day 63 and the average daily weight gain from days 0 to 63. This suggested that differences in growth performance between the CON group and the 0.8% SBOS group may be caused by changes in how nutrients were metabolized in the body.

### 4.2. Carcass Traits and Meat Quality

The results of this study showed that compared with the CON group, adding 0.8% SBOS to the diet significantly increased the carcass weight, dressing percentage, and carcass lean percentage of growing–finishing pigs, but decreased the average backfat depth. Therefore, we find that pigs with added 0.8% SBOS tend to be leaner. The main reason for the increase in carcass weight and dressing percentage may be that adding 0.8% SBOS reduces fat deposition capacity and increases lean meat deposition. The density of lean meat per unit volume is greater and the mass is greater. Although the addition of SBOS changed carcass traits, we found no significant effect on meat quality. No studies have been found on the impact of directly adding SBOS on the quality of meat in growing fattening pigs. However, a study has shown that adding white lupin (contains SBOS) to the diets of growing fattening pigs does not have any negative impacts on meat nutritional quality or carcass characteristics [25]. In short, the above results further indicate that the main reason why adding 0.8% SBOS to the diet increased the ADG and carcass traits may be due to the reduction in fat deposition. Besides, the outcomes indicated that the main reason why adding 0.8% SBOS to the diet improves ADG and carcass traits may be that the metabolic pathway changes after energy enters the body. The body uses more energy to synthesize muscle and reduces fat synthesis.

### 4.3. SCFAs, Fat Deposits, and Blood Profile

SCFAs are the intermediate and final products produced by the microbial fermentation of indigestible residues, such as polysaccharides, oligosaccharides, proteins, and peptides, in the mammalian gastrointestinal tract [26]. The fermentation process of carbohydrates provides the colon with metabolic end products and energy for the growth or maintenance of intestinal flora. Acetate primarily enters the portal system and serves as a peripheral energy source; propionate is partially metabolized by colonocytes and mainly by the liver; and butyrate is the most important fuel for colonocytes in humans and pigs. Rapid entry of fermentable substrates into the hindgut results in the production of lactic and succinic acids [27]. The results of this study showed that adding SBOS can significantly increase the content of acetate, propionate, and butyrate in feces. In addition, adding 0.8% SBOS to the diet was more effective in increasing the concentrations of acetate, propionate, and butyrate than in the 0.2% and 0.4% SBOS groups. This was consistent with the previous results of in vitro fermentation using SBOS as the substrate and colonic digesta as the inoculum, and we found that the production of acetic acid, propionic acid, and butyric acid increased [5]. We also measured blood components. Compared with the CON group, the results showed that the concentration of HDL-C in the blood of the 0.8% SBOS group significantly increased. This was consistent with the results indicating that adding SBOS to high-fat diets in mice can significantly increase HDL-C concentration and significantly improve dyslipidemia [28]. However, we did not find that the addition of SBOS improved the concentrations of T-CHO and TG as described in the above studies. Therefore, we can assume that adding 0.8% SBOS to the diet can increase lipid metabolism to a certain extent. Our study also found that adding 0.8% SBOS to the diet significantly reduced the enzyme activities of ME and FAS, but had no significant effect on the lipid content of adipose tissue and adipocyte diameter. FAS is a key multifunctional enzyme in fatty acid synthesis [29]. ME can decarboxylate malic acid to pyruvate and form NADPH, and plays a key role in the biosynthesis of fatty acids [30,31]. It was found that adding 0.8% SBOS to the diet can significantly reduce fat anabolism and lipid deposition. This also explained why pigs treated with 0.8% SBOS tend to be leaner in terms of carcass traits. In summary, adding 0.8% SBOS to the diet can increase the composition of fecal SCFAs, change the energy supply, increase lipid catabolism, and reduce fat anabolism, thereby affecting ADG.

## 5. Conclusions

The growth performance of growing–finishing pigs was not significantly affected by dietary supplementation with 0.2% and 0.4% SBOS. The addition of 0.8% SBOS to the diet altered SCFAs’ content, reduced fat deposition, and improved carcass traits, ADFI, and ADG.

## Figures and Tables

**Table 1:** Basal diets’ formulation and composition.

Items	Day 0–21	Day 22–63
Corn	72.65	80.90
Soybean meal	15.00	11.00
Wheat bran	8.00	4.00
Soybean oil	1.00	1.00
Dicalcium phosphate	1.20	1.00
Limestone	0.70	0.60
Salt	0.40	0.40
L-Lys.HCL, 78.8%	0.40	0.40
DL-Met, 98%	0.10	0.10
L-Thr, 97.5%	0.15	0.20
Choline chloride	0.10	0.10
Premix ^1^	0.30	0.30
Total	100.00	100.00
Nutrient content, %		
Digestible energy, kcal/kg	3393	3407
Crude protein	14.11	12.42
Ca	0.64	0.54
P	0.59	0.50
Ca: P	1.10	1.08

^1^ Premix provides per kilogram of feed: Vitamin A_5_ 512 IU, Vitamin D_2_ 250 IU, Vitamin E 24 mg, Vitamin K 3 mg, Vitamin B_12_ 0.024 mg, Riboflavin 6 mg, D-pantothenic acid 15 mg, Niacin 20 mg, Vitamin B_6_ 3 mg, biotin 0.15 mg, folic acid 1.2 mg, iron 100 mg, manganese 30 mg, copper 15 mg, iodine 0.3 mg, selenium 0.2 mg, and zinc 100 mg.

**Table 2:** Effects of dietary soybean oligosaccharides’ supplementation on growth performance of growing and finishing pigs.

Items	CON	Soybean Oligosaccharides, %			SEM	*p*-Values
		0.2	0.4	0.8		
Average body weight, kg						
Initial	52.56	52.58	52.50	52.32	0.105	0.292
Day 21	71.62	71.97	72.25	71.88	0.202	0.208
Day 42	93.33	94.35	93.84	94.32	0.751	0.747
Day 63	119.1 ^b^	120.3 ^ab^	119.3 ^ab^	122.5 ^a^	0.841	0.032
Average daily weight gain, g/d						
Day 0–21	907.7	923.3	940.1	931.8	10.43	0.177
Day 21–42	1034	1066	1028	1069	38.15	0.824
Day 42–63	1227	1236	1214	1340	53.64	0.335
Day 0–63	1056 ^b^	1075 ^ab^	1061 ^ab^	1114 ^a^	14.02	0.029
Average daily feed intake, g/d						
Day 0–21	2082	2151	2173	2133	25.90	0.106
Day 21–42	2855	2908	2894	2985	110.1	0.864
Day 42–63	3310	3340	3285	3642	148.1	0.304
Day 0–63	2661	2702	2671	2787	38.58	0.112
Feed–to–gain ratio						
Day 0–21	2.293	2.330	2.313	2.289	0.016	0.282
Day 21–42	2.760	2.729	2.813	2.796	0.025	0.113
Day 42–63	2.699	2.704	2.708	2.714	0.025	0.978
Day 0–63	2.520	2.513	2.519	2.504	0.024	0.960

Note: SEM, standard error of the mean. ^a,b^ Means the difference is significant for different superscripts in the same row (*p* < 0.05).

**Table 3:** Effects of dietary soybean oligosaccharides’ supplementation on nutrient digestibility of growing and finishing pigs.

Items, %	CON	Soybean Oligosaccharides, %			SEM	*p*-Values
		0.2	0.4	0.8		
Dry matter	90.13	90.63	90.50	89.75	0.285	0.150
Crude protein	81.83	81.51	82.57	82.01	0.329	0.162
Ether extract	72.62	71.95	72.96	72.49	0.295	0.127
Gross energy	84.01	84.38	84.06	83.93	0.229	0.531

Note: SEM, standard error of the mean.

**Table 4:** Effects of dietary soybean oligosaccharides’ supplementation on blood profile of growing and finishing pigs.

Items, mmol/L	CON	Soybean Oligosaccharides, %			SEM	*p*-Values
		0.2	0.4	0.8		
LDL-C	1.05	1.08	0.83	0.88	0.215	0.801
HDL-C	0.41 ^b^	0.43 ^b^	0.53 ^ab^	0.67 ^a^	0.046	0.002
GLU	4.68	4.54	4.60	4.41	0.163	0.694
T-CHO	1.49	1.48	1.46	1.33	0.117	0.740
TG	0.53	0.45	0.46	0.50	0.085	0.897

Note: LDL-C, low-density lipoprotein cholesterol; HDL-C, high-density lipoprotein cholesterol; GLU, glucose; T-CHO, total cholesterol; TG, triglycerides; SEM, standard error of the mean. ^a,b^ Means the difference is significant for different superscripts in the same row (*p* < 0.05).

**Table 5:** Effects of dietary soybean oligosaccharides’ supplementation on carcass traits of growing and finishing pigs.

Items	CON	Soybean Oligosaccharides, %			SEM	*p*-Values
		0.2	0.4	0.8		
Carcass weight, kg	77.21 ^b^	78.07 ^ab^	78.59 ^a^	79.07 ^a^	0.330	0.003
Carcass length, cm	107.1	107.4	106.9	108.1	0.852	0.773
Dressing percentage, %	64.75 ^b^	65.62 ^ab^	66.94 ^ab^	67.07 ^a^	0.586	0.026
Average backfat depth, mm	32.10 ^a^	31.54 ^ab^	31.73 ^ab^	30.29 ^b^	0.449	0.044
Carcass lean percentage, %	58.73 ^b^	59.58 ^ab^	59.78 ^ab^	62.48 ^a^	0.275	0.001

Note: SEM, standard error of the mean. ^a,b^ Means the difference is significant for different superscripts in the same row (*p* < 0.05).

**Table 6:** Effects of dietary soybean oligosaccharides’ supplementation on meat quality of growing and finishing pigs.

Items	CON	Soybean Oligosaccharides, %			SEM	*p*-Values
		0.2	0.4	0.8		
pH^45min^	6.44	6.45	6.51	6.28	0.100	0.443
pH^24h^	5.54	5.66	5.43	5.52	0.103	0.499
L*	44.05	44.51	43.99	44.32	0.223	0.332
a*	8.52	8.55	8.48	8.30	0.151	0.647
b*	6.34	6.65	6.61	6.44	0.122	0.260
Drip loss, %	2.11	1.95	2.08	2.05	0.117	0.808
Cooking loss, %	34.88	31.11	34.41	34.82	0.227	0.076
Shear force, N	9.56	8.99	9.43	9.40	0.178	0.149

Note: SEM, standard error of the mean.

**Table 7:** Effects of dietary soybean oligosaccharides’ supplementation on the composition of short-chain fatty acids in the feces of growing and finishing pigs.

Items, μmol/g	CON	Soybean Oligosaccharides, %			SEM	*p*-Values
		0.2	0.4	0.8		
Acetate	80.65 ^c^	90.90 ^b^	89.17 ^b^	102.9 ^a^	1.952	0.001
Propionate	16.84 ^c^	21.27 ^b^	20.15 ^b^	25.48 ^a^	0.390	0.001
Isobutyrate	15.89	16.12	15.70	15.36	0.291	0.222
Butyrate	15.24 ^c^	18.22 ^b^	17.16 ^b^	20.72 ^a^	0.495	0.001
Isovalerate	5.47	4.92	5.79	4.94	0.360	0.264
Valerate	7.63	7.19	7.28	7.42	0.147	0.199

Note: SEM, standard error of the mean. ^a,b,c^ Means the difference is significant for different superscripts in the same row (*p* < 0.05).

**Table 8:** Effects of dietary soybean oligosaccharides’ supplementation on the lipid metabolism of growing and finishing pigs.

Items	CON	Soybean Oligosaccharides, %			SEM	*p*-Value
		0.2	0.4	0.8		
Adipose tissue lipid content, %	71.21	72.03	72.10	72.46	1.328	0.923
Fat cell diameter, μm	58.58	60.82	60.07	60.27	1.591	0.780
Enzyme activity, nmol/(min g)						
ME	2985 ^a^	2898 ^a^	2708 ^ab^	2142 ^b^	153.7	0.003
G-6-PDH	744.0	750.3	766.6	749.8	30.76	0.959
FAS	259.6 ^a^	254.9 ^a^	216.0 ^ab^	150.9 ^b^	26.67	0.027

Note: SEM, standard error of the mean; ME, malic enzyme; G-6-PDH, glucose-6-phosphate dehydrogenase; FAS, fatty acid synthase. ^a,b^ Means the difference is significant for different superscripts in the same row (*p* < 0.05).

## Data Availability

The data presented in this study are contained within the article.
